# A Roadmap Towards Standards for Neurally Controlled End Effectors

**DOI:** 10.1109/OJEMB.2021.3059161

**Published:** 2021-02-12

**Authors:** Andrew Y. Paek, Justin A. Brantley, Akshay Sujatha Ravindran, Kevin Nathan, Yongtian He, David Eguren, Jesus G. Cruz-Garza, Sho Nakagome, Dilranjan S. Wickramasuriya, Jiajun Chang, Md Rashed-Al-Mahfuz, Md. Rafiul Amin, Nikunj A. Bhagat, Jose L. Contreras-Vidal

**Affiliations:** University of Houston 14743 Houston TX 77204 USA; University of Houston 14743 Houston TX 77204 USA; Department of BioengineeringUniversity of Pennsylvania 6572 Philadelphia PA 19104 USA; University of Houston 14743 Houston TX 77204 USA; Department of Design and Environmental AnalysisCornell University 5922 Ithaca NY 14853 USA; University of Houston 14743 Houston TX 77204 USA; Department of Computer Science and EngineeringUniversity of Rajshahi 118869 Rajshahi 6205 Bangladesh; University of Houston 14743 Houston TX 77204 USA; Feinstein Institutes for Medical Research 88982 Manhasset NY 11030 USA

**Keywords:** Brain-machine interface, exoskeletons, prosthetics, robotics, standards

## Abstract

The control and manipulation of various types of end effectors such as powered exoskeletons, prostheses, and ‘neural’ cursors by brain-machine interface (BMI) systems has been the target of many research projects. A seamless “plug and play” interface between any BMI and end effector is desired, wherein similar user's intent cause similar end effectors to behave identically. This report is based on the outcomes of an IEEE Standards Association Industry Connections working group on End Effectors for Brain-Machine Interfacing that convened to identify and address gaps in the existing standards for BMI-based solutions with a focus on the end-effector component. A roadmap towards standardization of end effectors for BMI systems is discussed by identifying current device standards that are applicable for end effectors. While current standards address basic electrical and mechanical safety, and to some extent, performance requirements, several gaps exist pertaining to unified terminologies, data communication protocols, patient safety and risk mitigation.

## I. Introduction

Brain-Machine or brain-computer interfaces (BMI/BCI) are systems that allow users to control devices or end effectors through their thoughts. End effectors such as exoskeletons and prostheses are often used for restoring, replacing or improving lost functionality caused by physical or neurological injury [1]. There is also a growing interest in using BMIs to control end effectors related to commercial and academic research projects [2]. Given the growing breadth of BMIs and end effectors, it is desired to have a set of standards that recommend how these systems should be linked with each other. Such a standard would greatly facilitate development by reducing the workload needed to make the systems compatible with each other. Also, as these systems become widely deployed for different medical conditions, standardization of these devices will become essential from a regulatory standpoint in order to demonstrate safety and efficacy.

Creating a device standard invokes a variety of considerations. For example, current BMI research related to restoring limb movements lay emphasis on the prediction of joint kinematics [3]–[7]. Many end effectors, such as powered prostheses and orthoses, operate with higher level commands (e.g., take a step forward), which may move multiple joints in a sequence. BMIs could also be used to control nonanthropomorphic end effectors such as a computer cursor's position, or a wheelchair's movement. Thus, the range of commands that can be sent to such a spectrum of external devices or end effectors by a BMI is likely to be large, so it could be argued that such a standard for end effectors should be limited in scope to promote innovation.

To address the lack of specific standards on neurotechnologies for BMI systems, in February 2020, the IEEE Standards Association Industry Connections released a roadmap focusing on standards for “*Neurotechnologies for Brain-Machine Interfacing*” [8]. The roadmap provided an overview of existing and ongoing standardization efforts with regards to different components of a closed-loop BMI system that ranged from sensor technology, end effectors, data storage and sharing, user needs, performance assessment and benchmarking. This paper is based on the end effectors’ section of the Standards Roadmap. We expand on the state-of-the art end effectors review presented in the Standards Roadmap [8] by adding the most recent studies. To make the review more relevant, we decided to limit the review to only those studies that demonstrate real-time control of end effectors using various neural interfaces. Next, we present relevant existing standards as well as the gaps therein. Finally, we conclude by summarizing some of the ongoing efforts and future directions towards standardization of neurally controlled end effectors.

This work does not comment on how a BMI system interfaces with its user or patient. The suggestions made here should apply to systems that use any kind of neural recordings, which can be noninvasive (e.g., scalp electroencephalography (EEG), etc.) or invasive (e.g., electrocorticography (ECoG), microelectrodes, etc.). Our discussion also applies to systems that use peripheral modalities such as limb motion kinematics, electromyography (EMG), or peripheral nerve recordings. Our suggestions should apply regardless of how neural features are mapped to the end-effector commands and what BMI algorithms are used. For example, if a robotic hand were controlled by a BMI that used either motor imagery, SSVEPs or other types of intent detection; all would yield standardized commands to either open or close the hand.

## II. Overview of State-of-the-art End Effectors

In the context of BMIs, end effectors can generally be considered as virtual and/or physical devices or objects (“things”) embedded with sensors, software and other technologies that would allow them to connect and exchange data with BMI systems. End effectors encompass a broad range of devices and functions, including physical or virtual devices/systems that assume both anthropomorphic and non-anthropomorphic forms. Current end effectors that are typically interfaced by BMI systems can be broadly divided into seven main categories, as described below. Here, we briefly review the state-of-the-art within each subcategory of end effectors.

### A. Upper and Lower Limb Powered Exoskeletons

Several upper limb powered exoskeletons have been developed primarily for rehabilitation of any combination of the shoulder, elbow, wrist or finger joints after spinal cord injury (SCI) and stroke. Two comprehensive reviews of these systems were recently published in 2017 [9], [10], with Stewart's review focusing specifically on hybrid exoskeletons, i.e., those which are used in conjunction with Functional Electrical Stimulation (FES) to facilitate muscle contraction [10]. Also, Gull *et al.* summarizes various design considerations for upper limb exoskeleton devices [11]. These exoskeletons utilize a variety of control strategies (e.g., assistance, correction and resistance based) [12] and can also serve as an end effector for BMIs, by taking advantage of neural signals as inputs such as EMG [4] and EEG [13], [14].

Lower limb powered robotic devices have emerged as assistive and rehabilitative tools, which enable individuals to walk and exercise in previously unavailable ways [15]. The devices fall under two categories: wearable joint actuators [16] or devices fixed to a platform (e.g., treadmill-based or paddle-based devices) [17]. Powered orthoses induce motion to one or more paralyzed lower limb joints using external power, usually via electric, pneumatic or hydraulic actuators [18], and have emerged as aids for over-ground, bipedal ambulation. The US Food and Drug Administration (FDA) has recognized exoskeletons as Class II medical devices with special controls [19]. Several studies have reviewed existing lower limb exoskeletons in a clinical context, evaluating the outcomes, effectiveness, possible benefits [20]–[23] and potential risks and adverse events [24]. Recent efforts to review and benchmark performance indicators found that the majority of studies are limited to straight walking performance review and lack the evaluation of tasks related to daily living [25], [26].

### B. Upper and Lower Limb Prostheses

For upper and lower limb amputees, motorized prosthetic devices can be interfaced with neurotechnology to help restore lost motor function. We refer the reader to the following reviews on upper limb [27], [28] and lower limb [29] prostheses available to amputees. Typically, most of these powered prostheses are controlled with surface electromyography, which detects motor intent through electric fields generated by engaged muscles [30]–[35]. There are ongoing developments to enhance myoelectric control through surgical interventions such as targeted muscle reinnervation [36], [37] and electrode implantation [38], [39]. Very few studies have demonstrated real time control of a potential upper limb prosthesis with BMIs, such as the control of hand shape with scalp EEG with amputees [40], and the online control of the grasping and opening of a robotic hand with MEG from paralyzed patients [41].

A major challenge associated with prostheses is the interface between the prosthesis and the residual limb. Traditional sockets present challenges for some individuals and can lead to discomfort and chronic skin problems, resulting in decreased mobility and lower quality of life [42]. Recent efforts have shown promise in overcoming these limitations through osseointegration, where the residual bone is surgically modified to serve as a mechanical anchor and data communication port to the prosthetic device.

### C. Robotic Manipulators

Robotic arms or manipulators can also be used to assist severely paralyzed individuals. These devices are not necessarily worn by the individual, and typically do not mimic the form of the natural human arm. They are usually designed to have multiple joints in the arm and manipulate objects through a claw-like gripper. While robotic arms are available for other contexts such as factory manufacturing, there is little discussion on how much clinical utility they may have for paralyzed patients. Despite this, robotic arms have been explored with BMIs, where individuals with tetraplegia were able to control them with implanted microelectrodes [44]–[47], ECoG [48], and scalp EEG [49].

### D. Functional Electrical Stimulation

Functional or Neuromuscular Electrical Stimulation (FES/NMES) is the application of brief electrical pulses using transcutaneous, percutaneous or implanted electrodes, in order to artificially contract the targeted muscles. FES systems have been widely used as a rehabilitative therapy and as an assistive device to restore lost motor function [50]. Typically, to control a FES device, the controller needs to specify stimulation parameters such as frequency, pulse-width, voltage/current output, and the specific channels to activate in order to evoke specific limb movements. Several studies have demonstrated BMI controlled FES systems for rehabilitation of stroke and SCI patients [51]–[55]. More recently, BMI controlled spinal cord stimulation that allows brain signals to bypass and electrically stimulate below the injury site have also been developed [56].

### E. Powered Wheelchairs

BMI-controlled powered wheelchairs provide augmentation and/or restoration of mobility. These devices have been used for research purposes, but currently there are no available BMI-controlled powered wheelchairs in the US market. Fernandez- Rodríguez *et al.* provides a review of the studies related to BMIs and wheelchairs [57].

### F. Virtual/Augmented Reality

Virtual Reality (VR) is a simulated environment that provides an immersive and interactive experience for the user. While VR immerses the user in a simulated environment, the Augmented Reality (AR) systems superimpose virtual elements in the real world thereby augmenting the view of the user in real-time. Virtual objects can vary from anthropomorphic objects, such as human avatars or limbs [58]–[60], to non-anthropomorphic objects and graphical user interfaces [61]–[65]. For a review of application of VR system in neuroscience research and therapeutics, readers are directed to Bohil *et al.* [66].

### G. Smart Physical Devices or Objects (“things”)

Recent efforts to connect BMI to objects through the Internet provide clear evidence for the coupling of these technologies into a ‘BMI-of-things’ (BMIoT) for consumer-based [67], [68] and healthcare applications [69], [70]. Data transfer protocols associated with BMI coupled with IoT include: Websockets [71], SYNAISTHISI [72], MQTT [73], HTTPS [70], and added security through blockchain [74].

Mobile neurotechnologies have been identified as a key sensing technology for the dynamic field of personalized healthcare systems, with unresolved standardized IoT architectures for neurotechnologies posing a significant challenge [75]. It is likely that other types of virtual and/or physical devices or objects (“things”) embedded with sensors, software and other technologies will be designed to allow them to connect and exchange data with BMI systems. This BMIoT could be valuable not only for individuals with disabilities, but also for able-bodied individuals to control home and office appliances, automobiles, workplace devices, and toys.

## III. Existing Standards for End Effectors

Table I presents a list of existing standards that are applicable to end effectors. This list was compiled by reviewing the state-of-the-art end effectors that are currently approved by the FDA and identifying the standards with which these devices were required to be compliant. In addition, standards currently under development or applicable to specific type of end effectors such as VR/AR and Osseointegrated implants, are all listed. Table I is not an exhaustive list but is meant as a reference for future manufacturers of end effectors that want to seek FDA approval or clearance, as well as to identify gaps that are relevant in the context of a neurally controlled end effector.

**TABLE I:** Current Standards Related to End-Effector Devices

		Standard			Description
**Electrical Specifications**		IEC 60601-1:2005+AMD1:2012, ANSI/AAMI ES60601-1:2005/(R)2012, IEC 80601-2-78:2019			General safety
		IEC 60601-2-10:2012+AMD1:2016			Stimulator safety
		IEC 60601-2-40:2016			EMG safety
		IEC 60601-1-2:2014, ETSI EN 301 489-1, ETSI EN 301 489-3, BS EN 50561-1:2013			Electromagnetic
		IEC 62304:2006+AMD1:2015			Software
		IEC 60601-1-10:2007+AMD1:2013			Closed-loop control
		ANSI/AAMI HA60601-1-11:2015			Devices for home healthcare
		ANSI/IEC 60529-2004			Electrical enclosure
		UL 1642 5th Ed.			Lithium Batteries
		ISO/WD 7176-14, ISO 7176-4:2008			Wheelchair power/controls
		IEEE 1856			Prognostics & Health Management of Electronic Systems
**Mechanical**		ISO 10328:2016, ISO 15032:2000, ISO 22675:2016, ISO/TR 22676:2006, ISO/TS 16955:2016, ISO 22523:2006 ,			Requirements and testing
		ISO 7176-6:2001, ISO 7176-2:2017			Wheelchair speed and dynamics
**General**		ISO 14971:2007			Risk management
		ISO 13485:2016, ISO 9001:2015			Quality management of medical devices
		ISO 15223-1:2016			Labeling
		ISO 10993-1:2009 , ISO 10993-10:2010 , ISO 10993-5:2009			Biocompatibility
		AAMI ANSI HE75:2009/(R)2013			Human factors engineering
		AAMI TIR49:2013			Instructional materials
		AAMI ANSI IEC 62366-1:2015			Application and usability
		ISO 14001:2015			Environmental management
		WHO standards for prosthetics and orthotics. Geneva: World Health Organization; 2017. License: CC BY-NC-SA 3.0 IGO.			Global standards for prosthetics and orthotics
**Definition & Terminology**		ISO 8548-1:1989 , ISO 8548-2:1993 , ISO 8548-3:1993 , ISO 8548-4:1998 , ISO 8548-5:2003 ,			Limb deficiencies
		ISO 8549-1:1989 , ISO 8549-2:1989 , ISO 8549-3: 1989 , ISO 8549-4:2014			Prosthetics & orthotics vocabulary
		ISO 8551:2003 , ISO 21065:2017 , ISO 29781:2008 , ISO 29782:2008			Functional deficiencies & rehabilitation
		ISO 29783-1:2008 , ISO 29783-2:2015 , ISO 29783-3:2016			Human gait
		ISO 13404:2007, ISO 13405-1:2015, ISO 13405-2:2015, ISO 21064:2017, ISO 21063:2017			Prosthetics & Orthotics components
		IEEE 1872-2015 , IEEE P1872.1 , IEEE 7007 , IEEE 7008			Robotics
**IoT**		IEEE 1451-99			Harmonization and Security
		IEEE 2413			Architectural Framework
			Standard	Description	
			IEEE 2510	Sensor performance & Quality	
**Virtual Reality & Augmented Reality**			IEEE 2048.1	Definitions	
			IEEE 2048.2 , 2048.3 , 2048.7 , 2048.8	Visual	
			IEEE 2048.9 , 2048.10	Audio	
			IEEE 2014.6	Interface	
			IEEE 3333.1.1-2015	User Experience	
			IEEE 2048.4	Person Identify	
			IEEE 2048.5	Safety	
			IEEE 2048.12	Content ratings	
**Implants (e.g., Osseointegrated Devices)**	**Materials**		ISO 5832-3:2016	Wrought titanium 6-aluminium 4-vanadium alloy	
			ASTM F136-13	Wrought titanium-6 aluminum-4 vanadium ELI (extra low interstitial)	
			ASTM F67-13	Unalloyed titanium	
			ASTM F899-12b	Wrought Stainless Steels	
	**Mechanical**		ASTM F88/F88M-09	Seal strength	
			ISO 10993-18:2005	Chemical Characterization	
			ISO 11607-2:2006	Forming, Sealing & Assembly processes	
			ASTM F1929-12	Detecting seal leaks	
	**General**		ISO 14971:2007	Risk Management	
			ISO 15223-1:2016	Labeling	
			ISO 14644-1:2015	Controlled Environments	
			IEC 62366-1:2015	Usability	
			BS EN 1041	Device Information

**TABLE I:** (*Continued*) Current Standards Related to End-Effector Devices

		Standard			Description
**Electrical Specifications**		IEC 60601-1:2005+AMD1:2012, ANSI/AAMI ES60601-1:2005/(R)2012, IEC 80601-2-78:2019			General safety
		IEC 60601-2-10:2012+AMD1:2016			Stimulator safety
		IEC 60601-2-40:2016			EMG safety
		IEC 60601-1-2:2014, ETSI EN 301 489-1, ETSI EN 301 489-3 , BS EN 50561-1:2013			Electromagnetic
		IEC 62304:2006+AMD1:2015			Software
		IEC 60601-1-10:2007+AMD1:2013			Closed-loop control
		ANSI/AAMI HA60601-1-11:2015			Devices for home healthcare
		ANSI/IEC 60529-2004			Electrical enclosure
		UL 1642 5th Ed.			Lithium Batteries
		ISO/WD 7176-14, ISO 7176-4:2008			Wheelchair power/controls
		IEEE 1856			Prognostics & Health Management of Electronic Systems
**Mechanical**		ISO 10328:2016, ISO 15032:2000, ISO 22675:2016, ISO/TR 22676:2006, ISO/TS 16955:2016, ISO 22523:2006 ,			Requirements and testing
		ISO 7176-6:2001, ISO 7176-2:2017			Wheelchair speed and dynamics
**General**		ISO 14971:2007			Risk management
		ISO 13485:2016, ISO 9001:2015			Quality management of medical devices
		ISO 15223-1:2016			Labeling
		ISO 10993-1:2009 , ISO 10993-10:2010 , ISO 10993-5:2009			Biocompatibility
		AAMI ANSI HE75:2009/(R)2013			Human factors engineering
		AAMI TIR49:2013			Instructional materials
		AAMI ANSI IEC 62366-1:2015			Application and usability
		ISO 14001:2015			Environmental management
		WHO standards for prosthetics and orthotics. Geneva: World Health Organization; 2017. License: CC BY-NC-SA 3.0 IGO.			Global standards for prosthetics and orthotics
**Definition & Terminology**		ISO 8548-1:1989 , ISO 8548-2:1993 , ISO 8548-3:1993 , ISO 8548-4:1998 , ISO 8548-5:2003 ,			Limb deficiencies
		ISO 8549-1:1989 , ISO 8549-2:1989 , ISO 8549-3: 1989 , ISO 8549-4:2014			Prosthetics & orthotics vocabulary
		ISO 8551:2003 , ISO 21065:2017 , ISO 29781:2008 , ISO 29782:2008			Functional deficiencies & rehabilitation
		ISO 29783-1:2008 , ISO 29783-2:2015 , ISO 29783-3:2016			Human gait
		ISO 13404:2007, ISO 13405-1:2015, ISO 13405-2:2015, ISO 21064:2017, ISO 21063:2017			Prosthetics & Orthotics components
		IEEE 1872-2015 , IEEE P1872.1 , IEEE 7007 , IEEE 7008			Robotics
**IoT**		IEEE 1451-99			Harmonization and Security
		IEEE 2413			Architectural Framework
			Standard	Description	
			IEEE 2510	Sensor performance & Quality	
**Virtual Reality & Augmented Reality**			IEEE 2048.1	Definitions	
			IEEE 2048.2 , 2048.3 , 2048.7 , 2048.8	Visual	
			IEEE 2048.9 , 2048.10	Audio	
			IEEE 2014.6	Interface	
			IEEE 3333.1.1-2015	User Experience	
			IEEE 2048.4	Person Identify	
			IEEE 2048.5	Safety	
			IEEE 2048.12	Content ratings	
**Implants (e.g., Osseointegrated Devices)**	**Materials**		ISO 5832-3:2016	Wrought titanium 6-aluminium 4-vanadium alloy	
			ASTM F136-13	Wrought titanium-6 aluminum-4 vanadium ELI (extra low interstitial)	
			ASTM F67-13	Unalloyed titanium	
			ASTM F899-12b	Wrought Stainless Steels	
	**Mechanical**		ASTM F88/F88M-09	Seal strength	
			ISO 10993-18:2005	Chemical Characterization	
			ISO 11607-2:2006	Forming, Sealing & Assembly processes	
			ASTM F1929-12	Detecting seal leaks	
	**General**		ISO 14971:2007	Risk Management	
			ISO 15223-1:2016	Labeling	
			ISO 14644-1:2015	Controlled Environments	
			IEC 62366-1:2015	Usability	
			BS EN 1041	Device Information	

## IV. Gaps in Existing Standards

### A. BMI Relevant Terminologies

Despite the existence of standard terminology for certain end effectors (e.g., prosthetics and orthotics in Table I), there is lack of clarity on terminologies related to the BMI control of these devices [8]. Below we discuss these confounding terms and propose definitions from a BMI's perspective.

#### 1) Active Versus Passive Systems

Traditionally, end-effector systems wherein the patient voluntarily drives the movement of the system and receives minimal assistance in performing the movement are referred to as active-assistance systems [76]. Systems that do not rely on the patient's voluntary input, or only passively assists them through the movement, are referred to as passive systems. Recently, the IEC 80601-2-78:2019 standard defined ‘active controlled’ systems as those in which control can solely be with the robot or shared with the patient or operator. This definition confounds with the definition of traditionally passive systems.

Therefore, for BMI-controlled end effectors, we propose that active systems imply those systems in which commands decoded by the BMI will be used to manipulate the end effectors, generally through electromechanical actuation (e.g., robotics, BMIoT), digital manipulation of the virtual environment (VR/AR), or electrical stimulation (e.g., FES).

#### 2) Continuous Versus Discrete Commands

A BMI system can send out two types of commands which we designate as continuous or discrete. Continuous commands are associated with states that continuously evolve with time and could apply to commanding an end effector's joint angle, position, velocity, force, etc. Discrete commands are associated with a finite number of states in an end effector. Examples include walking or stopping with a leg prosthesis, opening or closing a robotic hand, turning left or right in a wheelchair, etc. This convention can help guide how commands are generated from a BMI system and passed to the end effector. Continuous commands can be generated, for example, from regression-based models and output a range of values with defined intervals and boundaries. Discrete commands can be generated from classification algorithms and outputs a set of integers that are mapped to distinct states in the end effector.

#### 3) Initial or Zero State

The initial state of the end effector at its initial resting state must be specified within the standard. While the exact definition of the starting state is dependent on the specific end-effector configuration, it is important that end effector's state definition includes the initial state (e.g., end effector position, on/off, etc.) that follows a universal coordinate reference frame so that the correct transformation can occur between the BMI's output and end effector's next state.

### B. Nomenclature of Motor Functions

End effectors can assume numerous physical configurations, depending on the design and desired use of the device. In the case of anthropomorphic devices, the end effector attempts to replicate, restore, or augment a type of human motor function. Thus, a taxonomy of functions should be developed to provide a standardized language when considering the prescribed use of the device. For example, grasp patterns are a set of unique hand postures that allow a robotic hand to manipulate different objects [77]. This can be adapted from medical and anatomy literature but should carefully consider the definitions in the context of a robotic system.

### C. Omissions of Motor Functions or Degrees-Of-Freedom

For specialization applications (e.g., industrial work), or to reduce complexity and costs, anthropomorphic end effectors are often designed to have fewer degrees of freedom than human limbs. For example, hand-based prostheses are “underactuated” where the natural finger joints are mimicked as a bent solid material, or mechanically coupled to a singular motor so that they do not move independently [78]. These strategies should be defined explicitly to facilitate control and comparisons in device capabilities.

### D. Standardized Communication Protocols

The interconnection between a BMI's sensing and processing modules and the end effector requires the development of standards for data communication. Ideally, this communication standard may allow ‘plug-and-play’ settings where a BMI system can interchange functionally similar endeffectors and expect the same behavior (without need for redesign). To meet these requirements, the IEEE/ISO standard 11073 (Point-of-care medical device communication) could be adapted.

### E. Potential Risks in Powered Exoskeletons

Exoskeletons have inherent risks that are not fully investigated or mitigated as discussed in [23], [79]: First, shared control is typically used in exoskeletons, where user intent commands the end effector, while internal control algorithms act to implement control of the device [80]. There is a need to standardize how to prioritize commands from the user and the device to ensure safety during hazardous scenarios (e.g., falls, slips, etc.). Second, among the adverse events reported during use of an exoskeleton, skin and soft tissue breakdown is the most frequently occurring type [24]. While clinicians refer to a number of management techniques for musculoskeletal injuries [81]–[83], specific guidelines for preventing such injuries during use of an exoskeleton do not exist. Third, falls pose a significant risk to elderly individuals, especially when they are strapped in an exoskeleton. Studies often conclude that the risk of falls is low during use of a particular device simply because no falls were observed during experiments or they were caught by the harness/staff in [23], [24]. Clearly, this poses risks to the user.

### F. Sensory Feedback

End effectors are currently being improved with sensory feedback, which involves the integration of environmental sensors that can be used to present sensory information back to the user. This is mostly pertinent to hand based robotic devices, where proper grasp function calls for the user to have a sense of finger forces and object textures [84]. Tactile feedback includes various modalities such as vibration motors [85] or electrical stimulation [86], [87]. There is also ongoing development in stimulating peripheral nerves [39] and the brain to simulate sensory percepts [88], [89]. We omit an in-depth review of all sensory modalities in this work, but we emphasize that a standardized protocol between a BMI and an end effector should have bi-directional communication to accommodate motor commands and sensory information.

## V. Ongoing Efforts & Industry Insights

In addition to the standards activities of the IEEE Standards Association, several initiatives are currently underway in order to develop standards within the neurotechnology space. The Center for Devices and Radiological Health (CDRH) of the Food and Drug Administration (FDA) recognizes that patient preference information can inform the design of a medical device, including end effectors. Moreover, patient preference can be an input in the design of medical devices and clinical trials, and form part of the regulatory process for medical device evaluation [90]. To address the importance of patient preference information, the FDA has published draft guidance [91].

The IEEE Robotics & Automation Society is developing a standard for wearable robotics, with the focus being on devices for non-medical applications, such as military, construction, and industry [92].

An EU-funded project called *EUROBENCH* was launched in January 2018 with the aim of developing a benchmarking framework for robotics. It mainly focuses on bipedal machines (i.e., exoskeletons, prosthetics, and humanoids) [93]. Also, another EU-funded initiative: “*Inbots Inclusive Robots for a better society*” is focused on building a multidisciplinary community that work on aspects of responsible research and innovation paradigms for interactive robotics [94].

*Industry Perspective*: It can be argued that standardization can promote interoperability, compatibility, reliability, safety, and effective operations in a global scale. As a case study of the importance of standardization, the IEEE SA Industry Connections working group discussed the issue of standardization with Blair Lock (CEO of Coapt LLC), a developer of a myoelectric pattern recognition system that is potentially compatible with every prosthetic hand, wrist, and elbow, currently on the market. Excerpts from this discussion are presented in the Supplementary Materials.

The ongoing development of standards for wearable robotics (IEEE RAS), Internet of Things (IoT), and Neurotechnologies for BMI (IEEE Standards Association) are very encouraging. Future standardization efforts must prioritize unification of terminologies across multiple fields and end effectors, as well as harmonization and safety of end effectors, performance and quality. Importantly, specific requirements for measuring performance of systems that rely on shared control between the user and the device, must be addressed in future standards.

## Supplemental Materials

Excerpts from the discussion in Section V.
