# Supplementary material for: A Roadmap Towards Standards for Neurally Controlled End Effectors
Source: IEEE Open J Eng Med Biol. 2021 Feb 12;2:84–90. doi: 10.1109/OJEMB.2021.3059161 (PMC8979628; doi:10.1109/OJEMB.2021.3059161)
Supplement: Excerpts from the discussion in Section V. [file supp1-3059161.pdf]

Supplementary Materials
A Roadmap towards Standards for Neural Controlled End Effectors: Actuators and Feedback Devices

Andrew Y. Paek, *Member, IEEE*, Justin A. Brantley, *Member, IEEE*, Akshay S. Ravindran, *Student Member, IEEE*, Kevin Nathan, *Student Member, IEEE*, Yongtian He, David Eguren, *Student Member, IEEE*, Jesus G. Cruz-Garza, *Member, IEEE*, Sho Nakagome, Dilranjan S. Wickramasuriya, *Student Member, IEEE*, Jiajun Chang, Md Rashed-Al-Mahfuz, Md. Rafiul Amin, *Student Member, IEEE*, Nikunj A. Bhagat and Jose L. Contreras-Vidal*, *Fellow, IEEE*

I. Case Study: Coapt LLC

"Coapt is a neurotechnological company founded on focused and dedicated research to deliver modern myoelectric control for the benefit of users and clinicians alike." Coapt provides a control solution that employs advanced pattern recognition algorithms that predict motor commands from electromyography (EMG). Their devices are marketed to help enhance the control of upper limb prosthesis for amputees. We interviewed Coapt CEO Blair Lock, to learn about their experiences and challenges on how they developed their device, particularly on the aspect that their device is compatible with multiple upper limb prosthetic devices on the market. The following summary is based on a phone interview that Akshay Sujatha Ravindran from the University of Houston's laboratory for noninvasive brain-machine interface systems did with the CEO Blair Lock.

1) *State of the art in communicating with upper limb prosthesis?*

The perception that is prevalent in many cases is that conventional prosthetic devices have their own communication protocol and their own physical interface. However, most of the traditional control is performed in an analog fashion and is not too complicated to work with. Everyone is excited about the world of robotic prosthetics, however most of these are still archaic in their design of the electrical connections and how they communicate with one another.

2) *How difficult it is to work with existing communication protocols?*

A major part in making different devices to become compatible with one another is, understanding how each component works well with others. Typically, this problem can be addressed in multiple ways depending on the level of complexity and collaborations with third party device companies. In devices belonging to the lower end of the complexity scale, they do not require either side to do much work/collaborate, as its operation is relatively well understood. For devices belonging to the higher end of the scale, having a collaboration helps. Even though some of them have different communication protocols, pretty much all of them use existing digital standards and having these collaborations helps modify the system to run the API and in ensuring that they allow Coapt's system to communicate with theirs using a polished API (see Figure 1).

This does not necessarily mean that it is a straightforward plug and play model wherein the two systems can be interfaced without any modifications. At the clinician's side, there is typically a need for slight modifications to be made to the prosthetic device which do not fall under engineering level modifications and can be performed by the clinicians themselves. The interface from the Coapt company comes pre-configured from their office ready to communicate with the device of interest.

3) *What is the willingness of prosthetic companies to disclose the control strategies with Coapt?*

An extensive 30-year record of conducting clinical trials and academic publications have showcased their value before the prosthetic companies and has aided them in securing sufficient scientific backing. This encouraged the companies to permit Coapt to indulge in engineering their devices to make them compatible, after following different Non-Disclosure agreements or other legal arrangements.

A common control paradigm is not a single ended question. Companies might not necessarily be interested in adopting standards mainly due to economic factors. Making their devices interoperate with others would de-verticalize the market which does not really improve their business. That is, limiting interoperability restricts buyers from seeking device components from competitors to maintain or upgrade their devices. While interoperability might excite researchers, companies are not necessarily attracted to it.

4) *View on standardizing neurotechnology?*

According to Coapt CEO Blair Lock, prosthetics is not yet a domain that is in dire straits without standardization. Unlike other larger-volume consumer industries, this field of neurotechnology is not yet advanced or "smart" enough to procure standardization. Given the current state of technology, the lack of standards does not currently hinder endpoint users. They can work with clinicians and prosthetists to source the required components from the respective manufacturers and assemble these components together.

5) *Conclusion*

Consideration of standards, modularity and interoperability among the prosthetic device industry can be challenged by economic factors, level of collaboration among device makers, technology complexity and the market size. While researchers and emerging companies may benefit from standards, modularity and interoperability for the design of new devices with advanced functions, current device companies may feel the need for 'de-verticalizing' the market, which might affect their market.

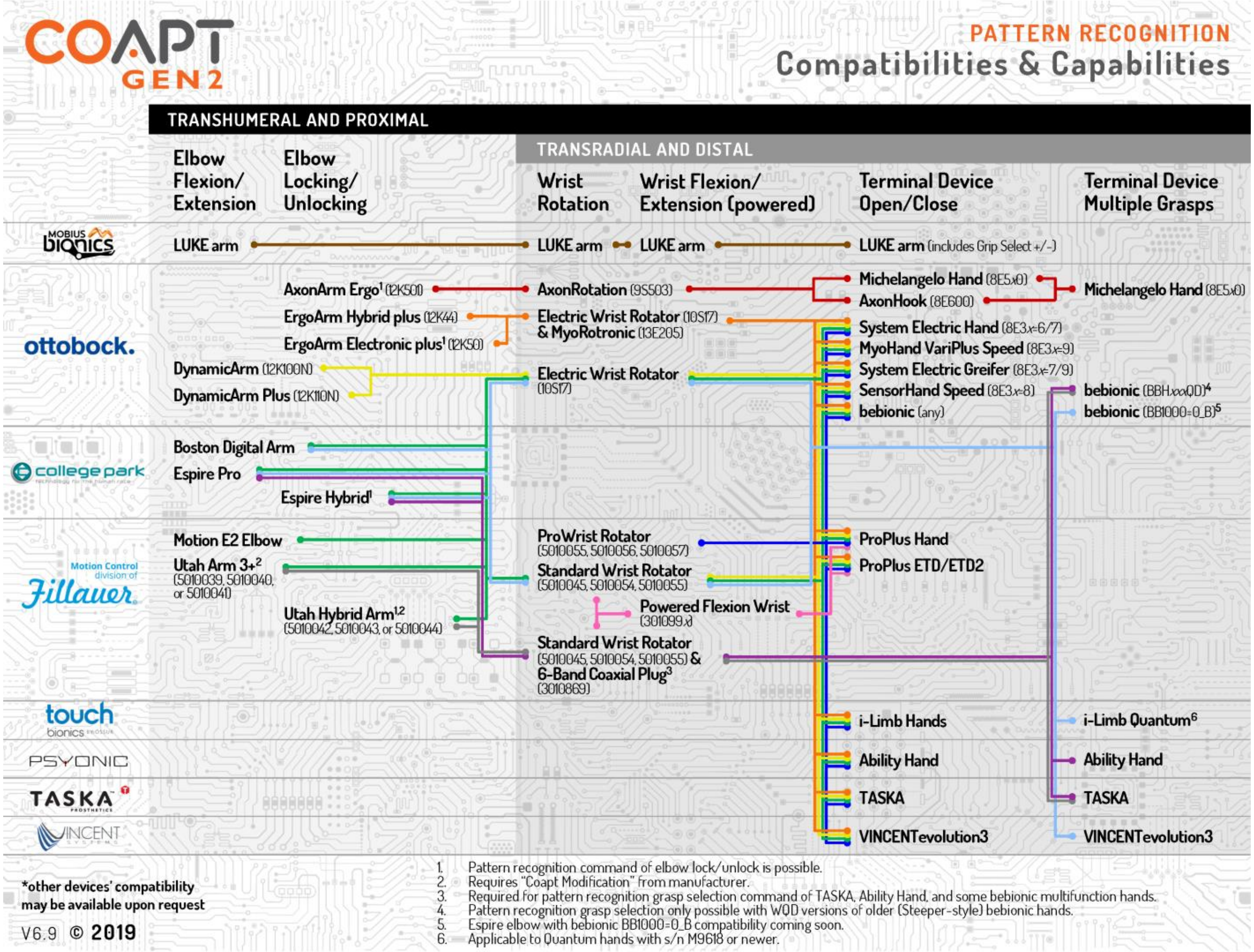

Figure 1) Configurations compatible with Coapt Gen 2 system.
